# Supplementary material for: Fusing subnational with national climate action is central to decarbonization: the case of the United States
Source: Nat Commun. 2020 Oct 16;11:5255. doi: 10.1038/s41467-020-18903-w (PMC7568566; doi:10.1038/s41467-020-18903-w)
Supplement: Supplementary file 1 — Supplementary Information [file 41467_2020_18903_MOESM1_ESM.pdf]

Supplementary Information

Table of Contents

1. Overview of Analytical Approach 2

2. Scenario Development 3

Survey of Climate Actions 3

Current Measures 4

Enhanced Non-Federal 4

Comprehensive 4

3. State Aggregation 5

Reference Scenario Analysis 5

Current Measures Analysis 5

Enhanced Policies Analysis 6

Avoiding Double Counting 7

4. Economy-Wide Analysis 8

Overview of GCAM-AP 8

Implementing Scenarios in GCAM 8

Assumptions and Sensitivity Analyses 9

5. Sector Descriptions 10

Coal Generation 12

Nuclear Generation 12

Gas Generation 12

Emissions Pricing and Caps 13

Renewable Energy 13

Oil and Gas Methane 15

Building Energy Efficiency 15

Building Electrification 16

Vehicles Miles Traveled Reductions 17

Vehicle Standards and Zero Emissions Vehicles 17

Hydrofluorocarbons 18

Biofuels 18

Industrial Emissions Reductions 19

Land Use 19

Other Methane and Nitrous Oxide 19

Supplementary References 20

Supplementary Methods

1. Overview of Analytical Approach

The paper analyzed the potential to reduce greenhouse gas (GHG) emissions in the U.S. through 2030 under three different scenarios for climate action:

- **Current Measures** projects where the U.S. is headed given current policies at national, state, and local levels.
- **Enhanced Non-Federal** examines outcomes under a broad expansion of cutting-edge climate policies on the part of states, cities, and businesses.
- **Comprehensive** explores a climate strategy integrating aggressive non-federal climate action with renewed federal engagement after 2020.

The analytical approach for modeling each scenario consisted of three steps. The first step was **scenario development** (Section 2), where we collected data on current and potential state, city, business, and federal climate actions. The second step, the **state aggregation** (Section 3), aggregated non-federal climate policies into state or regional activity data for each sector. The third step was an **economy-wide analysis** (Section 4), where we converted the results of the sectoral analysis into inputs for the U.S.-specific version of the Global Change Assessment Model (GCAM-AP, derived from GCAM-USA) and estimated economy-wide emissions.

A core feature of this analytical approach was the interaction between the state aggregation and economy-wide components (Supplementary Figure 1). Information from GCAM-AP served as an initial representation of activity levels for different sectors, such as electricity demand and generation, vehicles sales and vehicle miles traveled, and growth forecasts. This information was then processed and adjusted to represent the impacts of different climate actions within each sector. These impacts were then converted into sector-appropriate metrics at the state or regional level and incorporated into the economy-wide analysis using GCAM-AP.

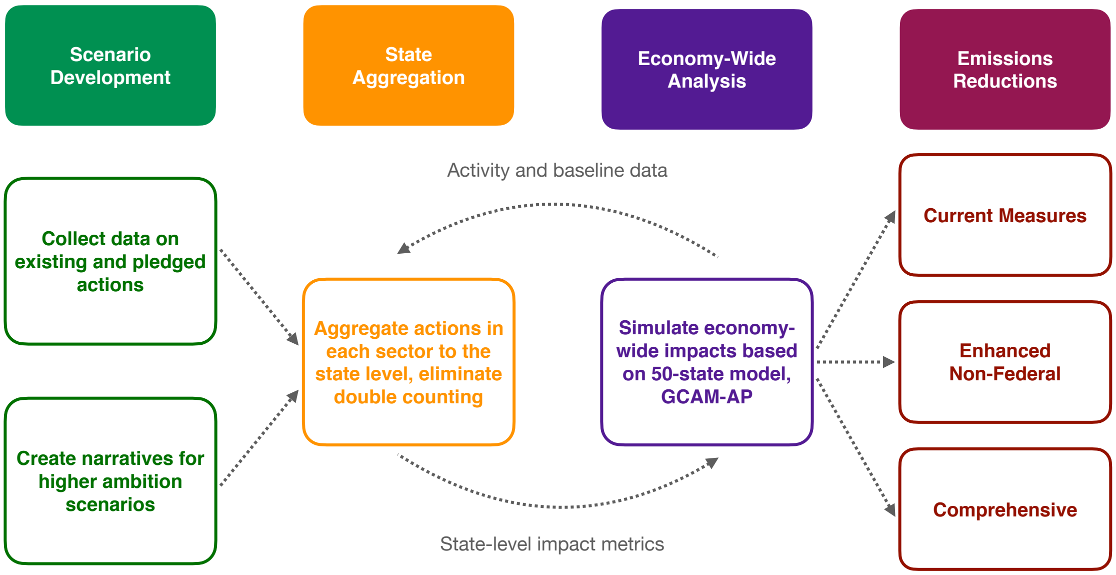

Supplementary Figure 1. Overview of Modeling Process.

2. Scenario Development

Survey of Climate Actions

We conducted a review of current climate policies and commitments at multiple scales as well as the potential for accelerated and expanded policies. Our approach to compiling and assessing the impact of city, state, and business actions was informed by existing protocols and methodologies such as the Non-State and Non-Federal Action Guidance developed through the Initiative for Climate Action Transparency[1], the Global Covenant of Mayors Emission Scenario methodology[2], and the Greenhouse Gas Protocol Mitigation Goal Standard and Policy and Action Standard[3]. We surveyed at a minimum all 50 states and the 285 most populous cities in the U.S. (i.e., those with a population over 100,000) and businesses that report relevant target information and/or activity data (including utility-level commitments[4] and voluntary emissions reductions from oil and gas[5] and refrigeration and air conditioning)[6]. For some sectors, additional cities beyond the 285 most populous were included due to availability of relevant data. We then identified a subset of quantifiable, high-impact actions for inclusion in the analysis and collected the necessary data (e.g., target information and historical activity levels) to quantify each action. This group included 34 states and 168 cities. Supplementary Figure 2 shows the geographic coverage of the subset of city and state actors with relevant actions identified climate action survey.

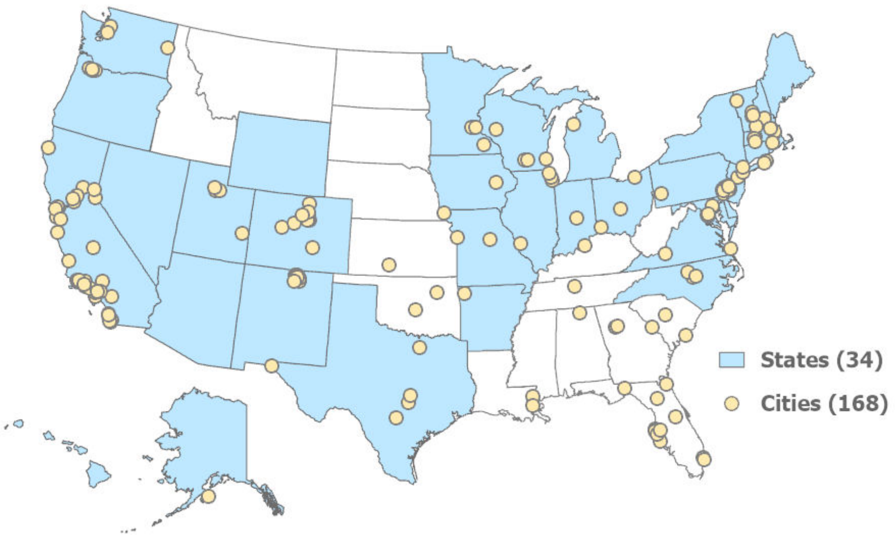

Supplementary Figure 2. Map of Actors with Current Policies or Commitments Quantified in the Analysis. *In surveying relevant non-federal climate policies, the analytic team identified a subset of high-impact, sector-specific policies ranging from state and local renewable electricity mandates or goals to regulations on HFCs and other non-CO_2 greenhouse gases. We identified 34 states and another 168 cities having at least one of these policies for inclusion in the analysis. Not shown are a number of business commitments also included in the analysis, including utilities with emissions reduction goals and corporate participants in EPA platforms such as GreenChill and GasStar. The approach for quantifying the impact of each of these measures is described in more detail, by actor group, in the sections below.*

The actions identified in the survey of climate policies and commitments described above served two primary purposes in the broader analysis. First, they served as direct inputs in the modeling for the Current Measures scenario of existing policies. Second, in cases of relatively ambitious or non-binding policies, they served as important benchmarks for potential scaled-up policy momentum for the Enhanced Non-Federal and Comprehensive scenarios. The overall contours of each of the three scenarios are further defined in the following sections.

Current Measures

Current state, city, and business climate actions differ in terms of concreteness and stringency, ranging from clearly defined, legally binding policies that are already in force to aspirational actions not currently enacted, but which would have significant impacts if achieved. Our Current Measures scenario includes actions that have been formally adopted by local and regional governments, are legally binding, and are currently being implemented. Additionally, we assume that current economic trends in the power sector will continue, leading to continued closure of coal plants beyond what other models, including the Annual Energy Outlook, assume in their reference case[7].

Enhanced Non-Federal

The Enhanced Non-Federal scenario includes many pledged actions that are not legally binding as well as a suite of new actions that could be put into place. This scenario projects how much a significant expansion of state, city, and business climate action could reduce GHG emissions, even without the help of the federal government. For this analysis, we grouped states into three tiers to reflect their willingness to act on climate. We identified these states by attributes such as membership in climate organizations, vocal leadership in support of climate action, ambitious emissions reduction targets or standards, and on-the-books climate policies. Tier 1 states are the *first movers*. In our scenario, these states adopt the most ambitious policies. States that are currently taking some measures to reduce emissions but not as quickly are categorized as Tier 2. These *fast follower* states implement some of the policies in the Tier 1 states but to a lesser extent. Finally, Tier 3 states do little with respect to passing climate policies.

- **Tier 1:** California, Colorado, Connecticut, Delaware, the District of Columbia, Hawaii, Illinois, Maine, Maryland, Massachusetts, Minnesota, New Hampshire, New Jersey, New Mexico, New York, Oregon, Pennsylvania, Rhode Island, Vermont, and Washington.
- **Tier 2:** Arizona, Iowa, Michigan, Missouri, Nevada, North Carolina, Ohio, Virginia, and Wisconsin.
- **Tier 3:** Alabama, Alaska, Arkansas, Florida, Georgia, Idaho, Indiana, Kansas, Kentucky, Louisiana, Mississippi, Montana, Nebraska, North Dakota, Oklahoma, South Carolina, South Dakota, Tennessee, Texas, Utah, West Virginia, and Wyoming.

Comprehensive

The Comprehensive scenario projects how much GHG emissions could be reduced with renewed and comprehensive engagement from the federal government, including new legislation that builds on the policies of the most ambitious states in the Enhanced Non-Federal scenario. In the Comprehensive scenario, after 2020 the Executive Branch and Congress implement a suite of new measures to decarbonize the economy. These new policies complement the efforts of states, cities, and businesses and fill in the gaps where federal policy is better suited. Our Comprehensive scenario requires expansive new policies and a massive buildout of new technologies and infrastructure.

3. State Aggregation

We developed a series of sector-specific calculations to integrate state, city, business, and federal actions and assess their net contribution at the state or regional level.[1] We first developed reference, no-policy baseline scenarios by sector using historic and projected data from EIA, EPA, and other sources and data from GCAM-AP. We then modeled the impacts of current policies as well as a suite of enhanced policies at the state or reginal level, taking into account overlapping impacts, relative to the sector and state-specific baselines.

Reference Scenario Analysis

Initial data from GCAM-AP was generally interpreted as a no-policy, reference scenario in which subnational policies, and some key federal policies, are not represented. Thus, the full impact of policies was applied to the baseline projections without needing to address overlap. Exceptions to this assumption and cases where any subnational policies were already embedded in the baseline are discussed in the sector descriptions in Section 5.

The GCAM-AP reference case scenario does include certain federal-level policies that have significant impacts within the sectors modeled. These include the federal production tax credit (PTC) and investment tax credit (ITC) in the renewable energy sector and federal fuel economy standards in the transportation sector. While our sectoral calculations typically represent the impact of state, city, or business policies only, final modeling results from GCAM-AP account for the combined impacts of these federal-level policies and the non-federal impacts from city, state, and business action. In addition, two types of federal policies not already included in GCAM-AP were explicitly modeled with non-federal actions before being passed back to GCAM-AP as inputs. These were the U.S. EPA Section 608 refrigerant management policy for HFCs and regulations to reduce fugitive emissions in the oil and gas sector.

Current Measures Analysis

Current state, city, and business actions differ in terms of concreteness and stringency, ranging from clearly defined, legally binding actions that are already in force to aspirational actions not currently enacted, but which would have significant impact if achieved. This poses a challenge in defining the policy scenarios. To address this, we categorized actions as one of two types: (1) existing actions, which have been formally adopted, are legally binding, and are currently being implemented and (2) pledged actions, which represent clearly defined intentions on the part of states, cities, and businesses, but which are not legally binding and may lack transparency on progress towards implementation to date. Only existing actions were included in the Current Measures scenario.

Supplementary Table 1 summarizes the current policies and commitments included in the analysis. The table specifically highlights actions that were modeled as being achieved by an explicit actor group (i.e., a city, state, or business). However, we also developed assumptions for certain policy sectors that were modeled in a more top-down fashion, such as future coal plant retirements and maintenance of the U.S. land sink, that do not correspond to a specific actor. While not included in this table, assumptions for these sectors were still incorporated into the broader scenario modeling and are described in the sections that follow. In some cases, policies at certain levels were not included due to data limitations, a lack of identifiable action at a given level (for example, no known city-level action on HFCs was identified), or a conscious decision on the part of the analytic team to exclude certain actions that lack specificity or stringency.

---

[1] Note that the term “sectors” is used to indicate policy areas in which state, city, and business impacts are explicitly modeled, such as renewable energy generation, vehicle miles traveled (VMT) reduction, or building and energy efficiency. The sectors described therefore do not necessarily correspond to traditional end-use sectors of the economy but rather types of policy interventions.

Supplementary Table 1. Policies Included in Current Measures

POLICY/ ACTION AREA	STATE	CITY	BUSINESS
Binding GHG Caps	Economy-wide carbon caps	Not included	Not included
Participation in Coalition Supporting Climate Action	Not included	Not included	Not included
Renewable Electricity Goals	RPS/CES	Renewable (RE) targets	Utility-level commitments
Oil & Gas System Fugitive Methane	New and existing equipment standards	Not included	Reductions reported through EPA Natural Gas STAR program
Nuclear Fleet Retention	Zero-emissions credit / nuclear fleet maintenance	Not included	Not included
Binding GHG Caps	Power sector specific GHG caps	Not included	Not included
Transportation	ZEV mandates, VMT targets	Municipal fleet targets, VMT targets	Not included
HFCs	HFC SNAP/ Phasedown Regulations and Refrigerant Mgmt. Programs	Not included	Reductions reported through EPA GreenChill program
Building & Industry energy demand	Energy Efficiency Resource Standards (EERS)	Energy efficiency targets	Not included

Enhanced Policies Analysis

While the Current Measures scenario was focused on existing policies, the Enhanced Non-Federal and Comprehensive scenarios were modeled based on analysis and assumptions by the study team about what policies or actions might be put into place and the best available data for what impact those actions might have. Context on how and why the study team selected certain actions and how they chose to model them is included in the sector descriptions (Section 5).

For the Enhanced Non-Federal scenario, states were grouped into three tiers (see Section 2) depending on current policies and historical willingness to lead on climate. These tiers are used for modeling purposes and intended to be illustrative. In reality, there is no bright line between states. Some states defined as Tier 2 or Tier 3 may take leadership-level actions in some sectors, and not all Tier 1 states will take the most ambitious actions across all sectors of the economy. Our tiered approach is intended to approximate the scale of action across all 50 states. Additionally, we did not use the tiered approach for all policies and sectors. For example, for coal retirements and the land sector, we used other metrics to model the aggregated impact of non-federal actions across the U.S.

The Comprehensive scenario projects how much greenhouse gas emissions could be reduced with renewed and comprehensive engagement from the federal government, including new legislation that builds on the policies of the most ambitious states.

Avoiding Double Counting

A key issue in aggregating city, state, business, and federal actions is how to feasibly estimate the impact of nested policies that may be overlapping or reinforcing in terms of impact. For example, a state may have a renewable energy target that is described in terms of a percentage of total generation to be provided by renewable sources. The state may also have a set of more granular policies or approaches that directly or indirectly contribute to achieving the target, such as tax credits, feed-in tariffs, infrastructure investment, or community choice aggregation legislation. For this analysis, we categorized climate policies and actions into policy levels. We defined Level 1 policies as those characterized by top-down targets such as a renewable electricity target for an entire state or city jurisdiction. We defined Level 2 policies as more granular measures contributing to the achievement of Level 1 policies. Our general approach was to estimate the impact of Level 1 policies rather than the Level 2 policies.

While top-down targets may often subsume more granular policies, this is not always the case. For example, significant infrastructure investment or wind and solar siting policies may occur within regions or communities without an on-the-books renewable electricity mandate and may nonetheless lead to increased renewable generation. Therefore, a limitation of our approach is that it does not capture the full impact of all possible actions. As the field of subnational policy modeling continues to develop, future analyses may build off of this approach and more explicitly model a fuller range of possible action. However, particularly when modeling impacts across multiple sectors of the economy, the inclusion of more granular, lower-level policies along with top-down targets can become exceedingly complex. Determinations of depth and breadth of the actions to be covered in the analysis ultimately depend on data and resource availability, the intended audience, and scope of work.

An additional challenge in aggregating state and local actions is that policies at the state, city, and business level overlap within a given action area. In this analysis, we first estimated the full impact of a given policy or action by each type of actor. We then aggregated the impact of these different actions at the state level. It is in this aggregation step that we factored out double counting where actions contributed to the same policy goal. This two-step approach allowed for flexibility in terms of attribution, so that the raw impact of actions at a given level (e.g., cities) can be assessed while controlling for double counting in the overall estimates. Assumptions regarding overlap vary by sector and are described in more detail in Section 5.

Supplementary Figure 3 presents an overview of the state aggregation process and an example of how this process works. We consider energy efficiency targets implemented at the state level as well as by cities within the state. In this example, a state has an energy efficiency target that would result in 1 TWh of energy savings. A city within the state also has its own energy savings goals. If the city's utility is excluded from compliance toward the state's policy, no overlap is assumed, and the aggregation step adds together both the city and state level impacts. However, if the city resides within a utility territory that must comply with the state goal, overlap is assumed to occur. In this case, we view the city's impact as contributing to the state's, and the aggregate total is equal to the state total. This example represents a simplified version of the approach and does not apply to all sectors. See Section 5 for a discussion of how overlap is assessed in each sector.

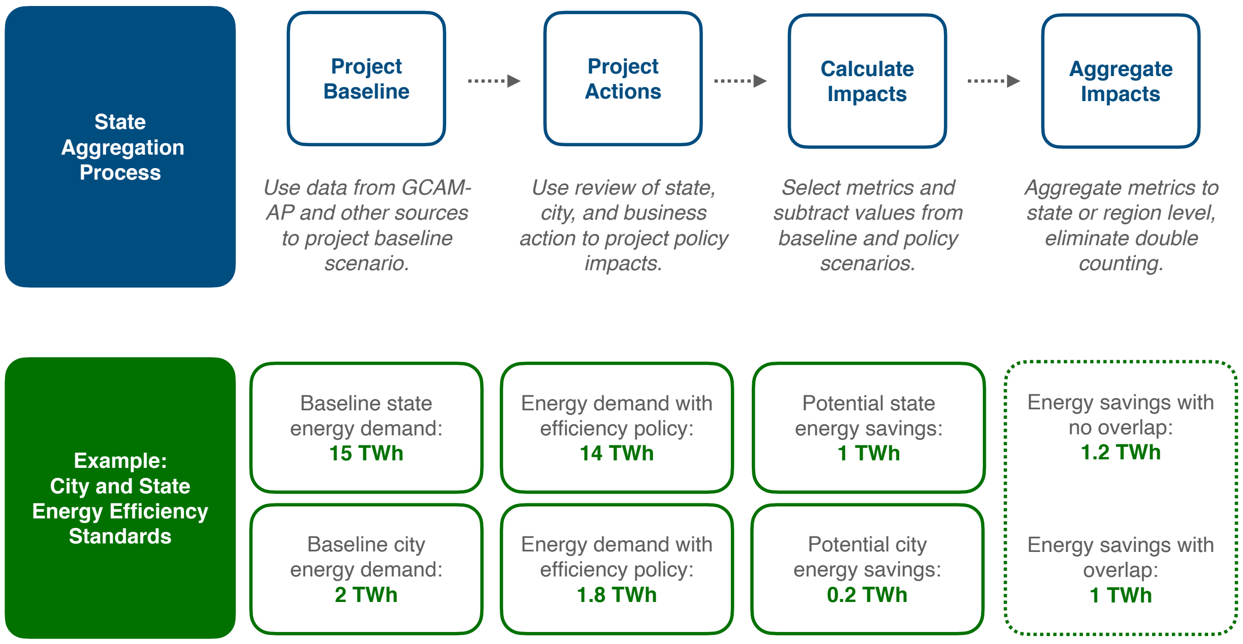

Supplementary Figure 3. State Aggregation Process and Energy Efficiency Example

The majority of overlap assumptions deal with the relationship between state- and city-level actions. While several corporate-level actions were included in our scoping analysis, only a select few were aggregated with state and city impacts and modeled in GCAM-AP. This approach results in part from a lack of reliable data on corporate actors to develop a meaningful methodology to account for overlap across all three levels of action (i.e., states, cities, and businesses). For many types of corporate action, available information does not specify the location (e.g., facility) where action was taken, making it difficult to include in a subnational analysis.

4. Economy-Wide Analysis

Overview of GCAM-AP

The estimates of economy-wide emissions use the results from the state aggregation and GCAM-AP. GCAM-AP is a version of GCAM-USA, which represents the energy and economy components of all 50 states and the District of Colombia. The energy system in GCAM-AP includes detailed representations of depletable primary resources as well as renewable resources such as bioenergy, hydro, solar, wind, and geothermal. GCAM-AP also includes representations of the processes that transform these resources to final energy carriers, such as refining and electric power. The electric power sector includes a range of technologies including those fueled by fossil fuels (with and without CCUS), renewables, bioenergy (with and without CCUS), and nuclear. Future improvements in technology costs and performance are represented exogenously over time. The market equilibrium in each 5-year period is solved by finding a set of market prices such that supplies and demands are equal to one another in all markets.[8] Further details on the GCAM modeling platform can be found on github.com/JGCRI/gcam-core.

Implementing Scenarios in GCAM

State, city, and business actions affecting energy-related CO_2 emissions were incorporated into the economy-wide analysis by directly incorporating inputs from the above-described state aggregation into GCAM-AP. Sectoral impacts

were converted into GCAM-appropriate inputs. Most sectoral metrics were aggregated at the state level; however, for some policies, the impacts were applied at the electricity grid region to allow for better consideration of the interactions among states, or at the national level due to data and model limitations. Emissions trajectories for certain non-CO_2 sources were calculated outside of GCAM-AP, including methane from oil and gas systems, coal mining, landfills, and livestock; nitrous oxide from croplands, livestock, and nitric and adipic acid production; and F-gases including HFCs. GCAM's non-CO_2 emissions inventory is based on the Emissions Database for Global Atmospheric Research (EDGAR) and differs from the inventories used in U.S. government analyses. For consistency, GCAM's non-CO_2 emissions outputs for 2005, 2010, and 2015 were normalized to historical values from the latest EPA inventory, and future emissions were scaled by the 2015 normalization factor. However, because recent research suggests that oil and gas methane emissions are approximately 60% higher than EPA inventories, these emissions were taken from a recent EDF meta-analysis.[9] The net CO_2 removal from natural and working lands in the U.S. was also calculated outside of GCAM-AP.

Assumptions and Sensitivity Analyses

The results of this analysis depend on many assumptions about how the U.S. and the world might evolve in the future. This includes, among many others, assumptions about economic activity, population growth, energy technology prices, and the degree to which natural lands in the U.S. are sequestering carbon. For this reason, estimating future GHG emissions cannot be considered a precise exercise. To help understand the possible range of outcomes and contextualize the results, we generated a range of sensitivity assumptions for important drivers (Supplementary Table 2). We focused on three sensitivities: population and economic growth, fossil energy prices, and the nature of the U.S. land use sink. While these sensitivities are not a full representation of all factors that might influence the aggregate implications of city, state, and business actions, they nonetheless provide insight into the range of possibilities and the level of certainty associated with the projections in this analysis. For comparison, these assumptions and sensitivities are compared against those in the EIA AEO and the BNEF NEO[10].

Supplementary Table 2: Assumptions and Sensitivities for Economy-Wide Analysis

Scenario	Current Measures Scenario	Sensitivity	AEO 2019 Comparison	BNEF NEO 2019 Comparison
**Economic Growth**	Overall GDP[1] growth at 1.9%/year	1.4%/year (low growth)<br>2.4%/year (high growth)	1.8%/year (reference)<br>1.2%/year (low growth)<br>2.2%/year (high growth)[6]	1.7%/year
**Population Growth**	Overall population[2] growth at 0.66%/year	0.56%/year (low growth)<br>0.86%/year (high growth)	0.65%/year (reference)<br>0.52%/year (low growth)<br>0.76%/year (high growth)[7]	0.66%/year
**Fuel Prices**	Oil prices grow 2.6%/year	1.4%/year (high resources)<br>3.6%/year (low resources)	2.4%/year (reference)<br>1.9%/year (high oil/gas)<br>3.0%/year (low oil/gas)[8]	--
	Gas prices grow 1.8%/year	0.6%/year (high resources)<br>2.8%/year (low resources)	2.0%/year (reference)<br>0.9%/year (low oil/gas) 4.3%/year (high oil/gas)[9]	2.0%/year
**Land Use**	Terrestrial carbon sink assumed to be largely unchanged relative to today	+/- 170 $MtCO_2e$[5]	--	--

**Electric Vehicles**	EV percentage of LDV sales in 2030:<br>Current Measures: 11%<br>Bottom Up: 61%<br>All In: 62%	Modeled as explicit policy measures	EVs make up 9.3% of LDV sales in 2030[10]	EVs make up 31.6% of LDV sales in 2030
**Solar Power**	Solar PV costs[3] drop 20% to $850/kW-DC by 2030 (Current Measures); 37% (All-In)	No Sensitivity	PV costs drop 13.8%[11]	Solar PV costs drop 37.1% by 2030
**Wind Power**	Wind turbine (class 5)[4] costs drop 18% to $1,225/kW by 2030 (Current Measures); 25% (All-In)	No Sensitivity	Wind turbine costs drop 9.6%[12]	By 2030, wind costs drop:<br>12.4% (low)<br>15.6% (mid)<br>25.6% (high)
**Coal Power Plant Retirements**	4%/year of capacity is retired	Modeled as explicit policy measures	3.4%/year (reference)<br>3.5%/year (high growth)<br>3.7%/year (low growth)[13]	6.3%/year

1. Notes: All future growth rates are for 2020 to 2030 unless otherwise noted. For comparison, the Congressional Budget Office projects 1.7% GDP growth [www.cbo.gov/publication/54918]
2. For comparison, the U.S. Census Bureau projects 0.66% population growth [https://www.census.gov/data/tables/2017/demo/popproj/2017-summary-tables.html]
3. Solar assumptions follow NREL ATB 2019 Medium Case
4. Wind assumptions follow NREL ATB 2019 Medium Case
5. Sensitivities in land use are based on the 2016 US Biennial Report calibrated to the latest emissions numbers and adjusted create symmetric sensitivity bounds.
6. AEO 2019, Table: Macroeconomic Indicators
7. AEO 2019, Table: Petroleum and Other Liquids Prices (Brent spot price)
8. AEO 2019, Table: Natural Gas Supply, Disposition and Prices (Henry hub spot price)
9. AEO 2019, Table: Light-Duty Vehicle Sales by Technology Type (Electric and Plug-In Electric Hybrid)
10. AEO 2019, Levelized Cost and Levelized Avoided Cost of New Generation Resources, Tables A1a, B1a (Levelized Capital Cost) - Percent change is interpolated from 2021 and 2040 data
11. AEO 2019, Levelized Cost and Levelized Avoided Cost of New Generation Resources, Tables A1a, B1a (Levelized Capital Cost) - Percent change is interpolated from 2012 and 2040 data
12. AEO 2019, Table: Electricity Generating Capacity

5. Sector Descriptions

This section provides a detailed, sector-by-sector overview of the policies included in our scenarios and the methods for quantifying these policies in the state aggregation and GCAM-AP. These policies are summarized in Supplementary Tables 3 and 4 for the Current Measures scenario and the Enhanced Non-Federal and Comprehensive scenarios.

Supplementary Table 3. Policies Included in Current Measures Scenario

Type of Action	Specific Measures Evaluated	Projected Impact if Achieved
**Renewable Mandates**	Binding Renewable Portfolio Standards in 28 states.	Renewable generation increases to 26% of total generation by 2030.
**Renewable Goals**	Significant non-binding renewable goals in 6 states, commitments in 142 U.S. cities, and recent renewable energy and/or decarbonization commitments from 24 utilities.	Not included in Current Measures scenario (achievement of these action is modeled in the Enhanced Non-Federal and Comprehensive scenarios)
**Retirement of Coal-Fired Power Plants**	Coal plants continue to retire according to announced and scheduled retirements and projected closures of additional uneconomic units.	Coal falls to 16% of total generation by 2030 from 27% in 2018.
**Nuclear Fleet Retention**	Policy action in Connecticut, Illinois, New Jersey, New York, and Ohio prevents at-risk plants from retiring.	Nuclear generation supplies 17% of generation in 2030.
**Regulation of Fugitive Oil and Gas Operations**	Regulations limit fugitive emissions through equipment standards for new or new and existing facilities in seven states. Federal standards to limit emissions from new facilities are also assumed to remain in effect, but at 75% effectiveness.	Cumulative 995 Mt CO_{2e} avoided emissions (2020-2030)
**Power Sector Carbon Caps**	Participation in Regional Greenhouse Gas Initiative (RGGI) by nine northeast states.	Cumulative 160 Mt CO_2e avoided emissions (2020-2030).
**Voluntary Mitigation of Fugitive Emissions from Oil and Gas Operations**	Voluntary mitigation actions on the part of oil and gas companies through EPA's GasStar to limit methane losses.	Not included in Current Measures scenario (achievement of these actions is modeled in the Enhanced Non-Federal and Comprehensive scenarios)
**Energy Efficiency Mandates**	Binding energy efficiency resource standards (EERS) in 20 states	Cumulative electricity savings of 1566 TWh and gas savings of 2360 BCF (2020-2030), or 541 Mt CO_2e in avoided emissions.
**Energy Efficiency Goals**	Non-binding standards in seven states and efficiency targets in 40 cities.	Not included in Current Measures scenario (achievement of these actions is modeled in the Enhanced Non-Federal and Comprehensive scenarios).
**Zero Emission Vehicle (ZEV) Mandates**	Current ZEV mandates in 10 states requiring minimum share of LDV vehicle sales to be zero emissions.	Cumulative total U.S. EV sales (BEV + PHEV) of 13.5 million (2020-2030), or 139 Mt CO_2e in avoided emissions
**Electric Vehicle Procurement Goals**	Procurement targets to electrify public fleets in 13 major U.S. cities.	Not included in Current Measures scenario (achievement of these actions is modeled in the Enhanced Non-Federal and Comprehensive scenarios).

**Vehicle Emissions Standards**	States and automakers adopt California's clean cars compromise ensuring incremental vehicle improvements through 2025.	New conventional cars achieve on-road efficiency of 42 miles per gallon by 2025 and remain at that level through 2030. New conventional light-duty trucks achieve 32 miles per gallon by 2025.
**Regulations to Mitigate HFC Emissions**	Regulations designed to phase down and replace HFCs with low-GWP alternatives in California, Vermont, and Washington and federal standards to limit leakage from refrigerants (EPA Sec. 608).	Cumulative 160 Mt CO_2e avoided emissions (2020-2030).
**Voluntary Mitigation of HFC Emissions**	Voluntary mitigation actions on the part of U.S. supermarkets to reduce HFC emissions through EPA's GreenChill program.	Not included in Current Measures scenario (achievement of these actions is modeled in the Enhanced Non-Federal and Comprehensive scenarios).
**Maintenance of Land Sink**	No specific actions evaluated for Current Measures scenario.	Land sink is assumed to remain at current levels (-714 Mt CO_2e) through 2030.

Supplementary Table 4. Policies Included in Enhanced Non-Federal and Comprehensive Scenarios

Policy	Enhanced Non-Federal Scenario 2030 Assumptions	Comprehensive Scenario 2030 Assumptions
**Clean Electricity**	States establish ambitious Clean Electricity or Renewable Portfolio Standard requirements. Tier 1, 2, and 3 states reach 60%, 40%, and 20% renewables, respectively. States prevent some at-risk nuclear plants from retiring.<br><br>2030 impact: Clean electricity provides 61% and renewable energy provides 40% of total national generation. Nuclear provides 17% of total national generation.	Federal clean electricity standard and tax incentives.<br><br>2030 impact: Clean electricity is 77% and renewable energy is 49% of total generation.
**Fossil Fuel Generation**	With most coal plants unprofitable, significant coal generation is phased out except in a few holdout states. Market trends and advocacy reduce coal generation nationally. Tier 1 and 2 states constrain new gas plant builds.<br><br>2030 impact: Coal produces 7% and gas without CCUS continues to provide 32% of total generation.	Federal policies result in near complete phaseout of coal generation by 2030 and cause gas generation to peak before 2025 and then decline.<br><br>2030 impact: Essentially no remaining coal generation; conventional gas produces only 23% of total generation. Gas with CUS produces 12% of generation.
**Oil and Gas Methane**	Tier 1 and 2 states adopt regulations covering new and existing sources, reducing fugitive methane emissions by more than 50%. Tier 3 states achieve reductions where policies are already in place or under development.<br><br>2030 impact: Oil and gas methane emissions are reduced by 34%.	Federal methane rules are reinstated and strengthened to cover new and existing sources. Fugitive methane emissions are reduced by 60% nationwide.<br><br>2030 impact: Oil and gas methane emissions are reduced by 60%.
**Buildings**	In Tier 1 and 2 states all new buildings are 100% electrified; policies are in place to ensure that almost all replacements of appliances from 2030 on are electrified. Tier 1 and 2 states enhance Energy Efficiency Resource Standards, with Tier 1 achieving 2% annual savings and Tier 2 achieving 1.5% annual savings.<br><br>2030 impact: Total direct emissions in buildings sector reduced by 28% compared to 2005.	Due to new federal standards and policies all new buildings are 100% electrified and replacement appliances from 2030 onward are electrified. Federal financing for residential and commercial retrofits. All states achieve further levels of energy savings where economic.<br><br>2030 impact: Total direct emissions in buildings reduced by 31% compared to 2005.
**Transportation**	Tier 1 and 2 states implement zero-emissions vehicle mandates and incentives. EVs (BEVs + PHEVs) reach 61% of light-duty vehicle sales. Heavy-duty electric vehicles comprise 15% of new sales in 2030 in Tier 1 and 2 states. In Tier 2 states, adoption is slightly lower. Federal rollbacks of LDV standards prove unsuccessful through 2025. Tier 1 and 2 states set ambitious new vehicle standards post-2026, improving internal combustion engine efficiency by 4% annually.<br><br>2030 impact: Total liquid fuel demand from transportation is down 21% from 2005 levels. Cumulative EV sales (2020-2030) reach 62 million vehicles nationwide.	Federal policies and standards promote zero-emissions vehicles so that nationwide EVs (BEVs + PHEVs) reach 62% of new light-duty vehicle sales and 100% of bus sales. The federal government reinstates the current LDV standards through 2025 and improves internal combustion engine efficiency 4% annually from 2026-2030. Furthermore, the federal government incentivizes the removal of old and inefficient vehicles from the road.<br><br>2030 impact: Total liquid fuel demand from transportation is down 22% from 2005 levels. Cumulative EV sales (2020-2030) reach 64 million vehicles nationwide.
**Industry**	Tier 1 states, and to a lesser degree Tier 2 and 3 states, incentivize industrial facilities to adopt best-in-class energy management practices and adopt electrified technology. States promote CCUS for industrial uses. Tier 1 and 2 states adopt policies to phase down HFCs and reduce leaks as agreed in the global Kigali Amendment. Tier 1 states adopt standards targeting cement emissions.<br><br>2030 impact: Total direct CO_2 emissions in the industrial sector are reduced to 5% below 2005 levels. HFCs and other fluorinated gases are reduced to 6% below 2005 levels.	Federal incentives lead all industrial facilities nationwide to adopt best-in-class energy management practices, and federal investments increase adoption of electrified technology. Federal policies and incentives promote adoption of CCUS. All states adopt policies to phase down HFCs and reduce leaks.<br><br>2030 impact: Total direct CO_2 emissions in the industrial sector are reduced to 7.5% below 2005 levels. HFCs and other fluorinated gases are reduced to 37% below 2005 levels.
**Land Use**	Tier 1 states and some Tier 2 states incentivize low-cost natural climate solutions such as natural forest management, optimal nutrient application, and the use of cover crops. All states mitigate agricultural methane and nitrous oxide emissions where it is cost effective.<br><br>2030 impact: Land carbon sink improves by about 80 Mt CO_2e, 11% higher than today.	Federal investments and incentives promote low-cost natural climate solutions nationwide. Strong federal incentives promote methane biodigesters to reduce methane from livestock.<br><br>2030 impact: Land carbon sink improves by about 167 Mt CO_2e, 23% higher than today. Livestock methane emissions reduced by 29% from reference case.
**Carbon Caps**	Tier 1 states meet their legislated economy-wide emissions reduction goals and partially meet their aspirational goals.<br><br>2030 impact: Emissions at the national level (all states) reduced 10%.	Tier 1 states meet their legislated economy-wide emissions reduction goals and fully meet their aspirational goals.<br><br>2030 impact: Emissions at the national level reduced 11%.

Coal Generation

The Current Measures scenario assumes that all coal units that have announced retirement plans will retire at their scheduled date through 2030 and also projects additional retirements from coal plants that are uneconomic. Specifically, for 2025, we assume that units in deregulated markets that had net negative long-run margins for at least five years between 2012-2017 close. The long-run margins were based on BNEF analysis in “Half of U.S. Coal Fleet on Shaky Economic Footing: Coal Plant Operating Margins Nationwide”[11]. By 2030, we assume that any unit in regulated markets with net negative long-run operating margins for all six years from 2012-2017 also close.

The Enhanced Non-Federal scenario assumes that in 2025, coal plants in deregulated markets that were uneconomic for 60% of the years since 2012 retire, while units in the regulated fleet retire if they have been uneconomic for 75% of the years. Our analysis assumed that 10 states that have historically been more protective of coal generation do not close any units by 2025. By 2030, all Tier 1 states, except Pennsylvania, completely retire coal generation. Units in unregulated markets that were uneconomic for half of the years since 2012 retire, and units in regulated utilities that were uneconomic for 60% of the years retire. Furthermore, the most uneconomic units in coal-protective states retire some of their coal. By 2030, 77 GW of coal generation remains in service. Our model assumes that bottom-up action prevents rebound in generation for the remaining units such that the average capacity factor for units falls from 53% today to 47% by 2030. This reduces coal generation to 317 TWh.

The Comprehensive scenario assumes ambitious federal policy building on state-level actions starting in 2021, with federal policies driving coal reductions in even the most resistant states. By 2025, capacity falls to 108 GW. By 2030, the U.S. removes all uncapped coal generation from the grid. Under this scenario, a small amount of coal capacity may remain in the system through 2030, where needed for spinning reserves or seasonal system balancing, but overall generation is minimal and any associated GHG emissions are captured and sequestered.

Nuclear Generation

In the Current Measures scenario, we assume 12.7 GW of at-risk capacity does not close due to existing policy actions in New York, Illinois, Connecticut, New Jersey, and Ohio. We assume that an additional 8.3 GW of other at-risk capacity does retire. The total amount of at-risk capacity was determined from information from the Union of Concerned Scientists and Beyond Nuclear accounting for every nuclear plant where a planned closure has been announced for before 2030. We also assume that the Georgia Vogtle Units currently under development come online in 2020-2021, adding 2.2 GW to the total U.S. fleet, according to DOE.[12]

In the Enhanced Non-Federal scenario, we assume that several other states enact policies to preserve an additional 2.6 GW of nuclear capacity. This is approximately half of the capacity that could be feasibly maintained if, for example, Pennsylvania and Iowa enacted policies to preserve their nuclear plants. It results in 95% of U.S. nuclear generation being preserved.

In the Comprehensive scenario, we assume that a federal clean electricity standard promotes the retention of nuclear plants and that 96% of nuclear generation is preserved, based in part on modeling analysis of the Clean Energy Standard Act of 2019 from Resources for the Future.[13]

Gas Generation

We developed plant-by-plant retirement assumptions for the Enhanced Non-Federal and Comprehensive scenarios using unit-level data on the existing gas fleet (from EIA’s Annual Electric Power Industry Report[14]) and a new RMI dataset from “The Growing Market for Clean Energy Portfolios: Economic Opportunities for a Shift from New Gas-Fired Generation to Clean Energy Across the United States Electricity Industry”[15]) of natural gas plants proposed for construction within the next four years.[16] The latter dataset also includes projections for the year in which the LCOE

for a new gas plant becomes more expensive than a comparable clean energy portfolio in each state (in some states, that threshold has already passed). We identified plausible reductions in planned construction and lifetimes of the existing fleet for each tier under the Enhanced Non-Federal and Comprehensive scenarios. For simplicity, fixed capacity factors based on turbine type were used, based on the overall capacity factors of the existing fleet.

The Enhanced Non-Federal scenario assumes a cessation of new gas builds in Tier 1 states due to both market competition and ambitious mitigation goals; constraints on new builds in Tier 2 states due to market competition; and business as usual new builds in Tier 3 states. In Tier 2 states, only planned gas facilities that are currently economic relative to a clean energy project are constructed. No new plants are built in Tier 2 states beyond those planned today. In Tier 3 states, all currently planned gas plants for the next four years are constructed, and plants continue to come online through 2030 at the same rate. Existing facilities across all tiers are retired at expected lifetimes for natural gas facilities (30 years for combined cycle, 43 years for others). Tier 1 and Tier 2 states retire plants slightly early but still within expected lifetimes. Based on these assumptions, gas generation remains essentially flat through 2030.

For the Comprehensive scenario, the assumptions were adjusted to reflect ambitious national efforts to deploy clean energy portfolios and phase out unabated fossil fuels. No new gas builds without carbon capture and underground storage (CCUS) are constructed in Tier 1 and Tier 2 states, and only new facilities that are currently economic are constructed in Tier 3 states. No new plants are built in Tier 3 beyond those planned today. The retirement age for existing facilities is assumed to be a little less than the historical average but largely still within the expected lifetime of gas facilities. Based on these assumptions, gas generation without CCUS declines by approximately 20 percent by 2030 relative to current levels. This scenario also assumes that many states incorporate gas with CCUS within their energy portfolios. This leads to roughly 517 GWh of gas with CCUS in 2030, or roughly 12% of overall generation.

Emissions Pricing and Caps

The Current Measures scenario includes the implementation of binding GHG emissions caps. For the purposes of this analysis, binding caps were limited to California's AB/SB 32 and the Regional Greenhouse Gas Initiative (RGGI) for states in the U.S. Northeast. This scenario does not include a significant number of economy wide GHG targets which have been recently adopted at the state level. These targets are included in the Enhanced Non-Federal and Comprehensive scenarios. We assumed participating RGGI states achieve a 30% reduction in power sector CO_2 emissions by 2030, relative to 2020 levels. We assumed California's 2030 target of a 40% economy-wide reduction in emissions from 1990 levels is fully achieved.

In the Enhanced Non-Federal scenario, we assumed that twelve states (in addition to California) that have GHG targets codified in legislation fully achieve their goals by 2030. For goals where the target year is after 2030, we assumed that states achieve linear progress toward these goals from the target base year through 2030. We also assumed that six states with more aspirational goals (e.g. those promulgated through executive orders but not yet codified in legislation) achieve 75% of their goals by 2030, or are on track to meet 75% of the goal if the target year is after 2030.

For the Comprehensive scenario, we assumed that complimentary federal policy leads to the achievement of all binding and non-binding state level GHG targets. In other words, all 19 states with targets fully achieve their targets by 2030, or are on track to fully achieve their targets if the target year is after 2030.

Renewable Energy

Our analysis includes 28 renewable portfolio standard (RPS) policies currently mandated by U.S. states and the District of Columbia. The renewable electricity generation driven by these policies was estimated for both hydroelectric and non-hydroelectric sources. We used state-level electricity load forecasts and effective RPS demand rates (percentage of electricity load to be supplied by renewable generation) derived from Lawrence Berkeley Lab

analysis in order to produce these estimates. [17] We obtained baseline state-level electricity sales data from EIA for the years 1990-2017.[18] State-level electricity load projections through model year 2030 were then calculated using annual growth rates from GCAM's state electricity demand outputs.

For city commitments, we estimated the impact of 144 current renewable electricity targets. Impact was quantified in terms of renewable energy demand (in GWh), derived from city-level electricity load forecasts and projected generation by fuel type. City-level targets were categorized as pledged actions and therefore included only in the higher ambition scenarios. We obtained city-level electricity load estimates from the National Renewable Energy Laboratory's State and Local Energy Data (SLED) tool.[19] SLED electricity consumption estimates by city (in MWh) for the year 2013 were projected forward through 2017 using state-level electricity consumption growth rates from EIA.[20] The city-level consumption estimates were then projected forward through model year 2030 using growth rates from GCAM's state electricity demand outputs. We assumed that a city's percentage of renewable energy increases linearly until 100% of the goal is reached in the target year.

We also assessed commitments to decarbonize electricity supply and increase the share of renewable generation on the part of 31 utilities. These targets were categorized as pledged actions and included only in the higher ambition scenarios. Data on utility-level commitments were compiled by WRI and also sourced from a Utility Carbon Reduction tracker developed by Smart Electric Power Alliance (SEPA).[21] We quantified the impact of these commitments by estimating baseline renewable generation levels for each utility's total present-day fleet and then assuming that its goals will be met through directly increasing the level of renewable energy generation relative to fossil fuel sources in the fleet. We calculated the baseline utility generation mix using data on plant-level assets from the US Plant Stack dataset curated by Bloomberg New Energy Finance (BNEF).[22] For companies that framed goals in terms of emission reductions, we assumed these reductions would be met by replacing fossil fuel generation with renewable energy sources.

We aggregated renewable energy generation resulting from the state, city, and utility actions described above at the state level, partially accounting for overlap across the three different levels of action. We accounted for city policies using a net percentage approach, where additional renewable demand from city goals in percentage terms (beyond the percentage required by state policies) is added to the state total. For example, a city with a 50% goal in a state with a 40% RPS add a net 10% renewable generation to its electricity load. Alternatively, if the city's projected renewable target were less than the state's 40% target, no additional renewable demand would be included. We applied the same net percentage approach when accounting for utility targets.

For the Enhanced Non-Federal scenario, we assumed that Tier 1 states achieve at least a 60% renewable portfolio standard by 2030, consistent with the most ambitious state policies currently in place. Tier 2 states enhance their ambition, achieving at least a 40% renewable portfolio standard by 2030. Finally, Tier 3 states achieve a 20% renewable portfolio standard by 2030. In rare cases where renewable penetration rates quantified in the Current Measures scenario exceeded the state tier rates, the current measures rates were carried over.

The Comprehensive scenario assumes all states, particularly in the Tier 3 category, further increase renewable penetration rates to at least 35%. Combined with assumptions on coal retirements, natural gas generation, nuclear, and CCUS, clean energy generation in the United States reaches 75% by 2030. The Enhanced Non-Federal and Comprehensive scenarios also assume that all existing and pledged actions are fully achieved, accounting for overlap using the same approach applied to the Current Measures scenario.

Oil and Gas Methane

We obtained data on state level emissions from oil and gas systems and projected reductions under no policy and multiple federal and state policy scenarios from the Environmental Defense Fund (EDF). We first quantified impacts of federal regulations at the state level. However, given uncertainty in the future legal status of federal oil and gas regulations including the EPA-issued New Source Performance Standards (NSPS), we assumed only 75% of the reduction potential of these regulations is achieved in the Current Measures scenario. In addition, states with policies already in place that match or exceed the emissions reduction potential of the federal rules were assumed to achieve 100% of the reduction potential within their jurisdictions in the Current Measures scenario. These states included California, Colorado, Pennsylvania, Utah, Ohio, and Wyoming.

For the Enhanced Non-Federal scenario, we assume that ambitious standards continue to move forward. Specifically, we assume that all states with aspirational regulations currently under development are able to achieve their goal. Data on the impacts of current aspirational state policies were provided by EDF. Beyond the achievement of aspirational regulations, we further assume that Tier 1 and 2 states achieve minimum reductions in oil and gas methane of 60% by 2030, based on the impact of best-in-class comprehensive regulations currently under development.

For the Comprehensive scenario, we further assume that by 2022, full compliance toward federal standards is achieved across all jurisdictions, reflecting restoration federal rules. We also assume that by 2030, all states achieve minimum reduction in oil and gas methane of 60% by 2030.

In addition to state and federal standards, we estimated the impact of voluntary commitments on the part of natural gas companies to reduce methane emissions through EPA's Gas STAR program and included these in the higher ambition scenarios.[23] We assumed that historic Gas STAR reductions reported by EPA would continue and increase proportionally with projected increases in oil and gas production activity, derived from EIA's AEO projections. We used emissions data from EPA's Facility Level Information on Greenhouse Gasses Tool (FLIGHT) to allocate estimated Gas Star reductions at the national level to states based on proportional share of emissions by segment (e.g. production, transmission, distribution).

Building Energy Efficiency

In the Current Measures scenario, we included the impact of 20 binding state-level energy efficiency resource standards (EERS) currently in place. These policies establish energy savings targets for electricity and/or natural gas demand that regulated entities within the state, such as utilities, are required to achieve. We obtained historic state-level commercial, residential, and industrial electricity and natural gas demand from the EIA for the years 1990-2017[24,25] and estimated annual demand projections through 2030 using growth rates from GCAM's state electricity and natural gas demand outputs. To quantify the projected impact of current EERS policies, we applied state-level average annual incremental electricity and/or natural gas savings targets as estimated by ACEEE's 2018 State Energy Efficiency Scorecard[26] to the state's projected demand. Because standards do not always apply to all energy sales within a state, we adjusted energy savings by the percentage of electricity or natural gas sales covered by the target. For state energy efficiency targets with specified end dates, we assumed that incremental energy savings would still be realized through the average measure lifetime as reported to EIA by utilities located in the state.[27]

For the Enhanced Non-Federal and Comprehensive scenarios, we assumed the achievement of state-level Energy Efficiency Resource Standards leading to annual energy savings of 2% in all Tier 1 states and 1.5% in all Tier 2 states in the Enhanced Non-Federal scenario. These targets were applied as minimums, and thus if higher levels of savings were achieved in a given state in the Current Measures scenario, these savings were retained.

In the Enhanced Non-Federal and Comprehensive scenarios, we also added several aspirational goals at state and city levels and assumed they were fully achieved. These included 7 non-binding state EERS policies that are limited by a cost-cap or allow certain groups of customers to opt-out of the program. In addition we assessed the impacts of 40 city-level energy efficiency targets. Data describing these targets were sourced from ACEEE and individual city government websites and climate actions plans.[28] Baseline electricity and natural gas demand data was obtained from the NREL's SLED tool[29] for the year 2013 and projected forward through 2017 using state-level demand growth rates from EIA. The city-level consumption estimates were then projected forward through model year 2030 using growth rates from GCAM's state demand outputs. For cities that have targets which apply only to certain sectors, the target was applied to the proportion of energy demand for that sector. Energy savings resulting from city energy efficiency targets were summed up to the state level and compared to the totals resulting from state-level action. This analysis assumes that 100% of the savings from a city target is additional if the city is served by a municipal utility that is exempted from the state's EERS, but that only 25% of the savings associated with a city target is additional if the city is served by an investor-owned utility that must comply with the state EERS.

For the Comprehensive Scenario only, we also assume that funding for the federal Weatherization Assistance Program (WAP) returns to the levels seen under the American Recovery and Reinvestment Act (ARRA), resulting in a commensurate increase in energy savings of approximately 5,341,628 MMBtu nationwide each year beginning in 2022.[30] We incorporated these savings into our analysis by taking the average electricity and natural gas savings by home type using data from Oak Ridge National Laboratory (ORNL)[31,32,33] and weighting it by home type based on the percentage of units that were served under WAP under ARRA. These savings compound as more homes are weatherized under the program each year through 2030.

Finally, for new buildings, we assumed that states continue to adopt new, more efficient codes at the approximate rate they have historically in the Enhanced Non-Federal and Comprehensive scenarios. We used results from a Pacific Northwest National Laboratory report[34] using this methodology for commercial and residential codes. We also modeled the impact of codes adopted by leading cities beginning in 2022. We applied these assumptions to major cities in Tier 1 states and additional cities in Tier 1, 2, or 3 states that have demonstrated climate leadership by either having a 100% renewable electricity goal[35] or being a member of the Climate Mayors initiative.[36] For these cities, we assumed that – after first calculating the impact of EERS standards and buildings codes at the state level – an additional 11.2% energy savings are achieved for the portion of each of these cities' energy demand from new or altered building stock each year. The 11.2% figure is the additional site energy savings attributed to New York's 2016 Stretch Code Supplement relative to ASHRAE 90.1-2013, according to a 2018 Pacific Northwest National Laboratory (PNNL) study.[37]

Building Electrification

The Enhanced Non-Federal scenario also assumes that space and water heating electrify in locations with greatest interest and economic incentive. We assume Tier 1 and 2 states achieve penetration rates of electric appliances consistent with NREL's high electrification scenario from "Electrification Futures Study: Scenarios of Electric Technology Adoption and Power Consumption for the United States"[38], while Tier 3 states achieve sales percentages in line with NREL's medium electrification scenario. We assume that states already seeing or approaching electrification levels projected by the NREL high scenario for 2030 achieve NREL's high electrification rates for 2050 by 2030. For the Comprehensive scenario, we assume all states achieve electric appliance sales in line with NREL's high scenario, except for the high-penetration group of states which achieve the higher 2050 standard. As electricity generation gets cleaner, electrified heating and cooling exhibit a stronger signal in total emissions reductions.

Vehicles Miles Traveled Reductions

The Current Measures scenario includes three state vehicle miles traveled (VMT) reduction targets (California, Vermont, Washington). We obtained information on these targets from ACEEE[39] and data on historical VMT from the DOT's FHWA Highway Statistics.[40] Baseline VMT growth rates by vehicle class, including the impacts of current federal policies (e.g., CAFE Standards), were estimated using GCAM, and state policies were added for the relevant vehicle categories.

We also assessed the impact of 15 city-level VMT reduction goals in the Enhanced Non-Federal and Comprehensive scenarios. Data describing these goals were sourced from ACEEE, city government websites, and published climate action plans.[41] City-level VMT baseline data were obtained from the NREL SLED tool[42] for the year 2013 and projected forward using GCAM state-level outputs. Our analysis conservatively assumes that a city-level VMT target would only be additional to the impact of a target within the state if the estimated decrease in VMT exceeds the state level goal in a given model year. For city VMT targets occurring in states without targets, 100% of the impact was included in the analysis.

For the Enhanced Non-Federal scenario, we also assume that Tier 1 states that do not already have VMT goals implement smart-growth programs that reduce VMT 2% by 2030 relative to baseline levels. The Comprehensive scenario assumes that VMT are reduced 2% by 2025 and 3.25% by 2030 relative to baseline levels nationwide.

Vehicle Standards and Zero Emissions Vehicles

The Current Measures scenario assumes that existing vehicle standards (for light-, medium-, and heavy-duty vehicles) and zero emission vehicle (ZEV) programs remain operative, despite uncertainty created by the current administration's actions, and that all states and automakers align with the California compromise on vehicles standards, achieving 3.7% improvements annually.[43] The Current Measures scenario also includes the impact of the California's Zero Emissions Vehicle program and the ten additional states that have signed on to the regulation.[44] Baseline plug-in hybrid electric vehicle (PHEV) and ZEV sales are calculated by taking EIA's AEO historic and projected vehicle sales through 2030 and disaggregating to individual states using vehicle registration totals published by FHWA.[45,46] Estimates of BEV and PHEV sales that would be additional to baseline sales through 2025 were derived from analysis conducted by EIA.[47] For years beyond 2025, it was assumed that BEV and PHEV sales percentages in ZEV mandate states would continue to increase in line with annual growth rates derived from AEO.

We also assessed the impact of eight quantifiable city electric vehicle procurement goals for the light-duty vehicle sector and included these in the Enhanced Non-Federal and Comprehensive scenarios. Data describing the city-level fleet procurement goals were sourced from ACEEE, individual city government websites, and published climate action plans.[48] We assumed a linear trend in cumulative procurement from the year enacted to the target year. Once the target year is reached, we assume that the cumulative total number of EVs will be maintained by the city through 2030. Our analysis conservatively assumes that city-level procurement targets occurring within ZEV mandate states would contribute to the state-level targets and result in no additional EVs on the road beyond the state goals. City level targets occurring in states without ZEV targets were assumed to be additional to the EV sales baseline.

For the Enhanced Non-Federal scenario, we also assume that California and Tier 1 and 2 states move forward with ambitious light-duty standards from 2026-2030, reducing carbon dioxide emissions from internal combustion engine (ICE) cars and light-trucks by 40 grams per mile by 2030. By 2030, new ICE cars achieve on average 48 miles per gallon (on-road tailpipe) and light-trucks achieve 34 miles per gallon. The Comprehensive scenario assumes the Obama era light-duty vehicle standards remain enforced and in place at their original stringency through 2025. After 2025, all light-duty vehicles improve GHG performance by 40 grams per mile, reaching 55 miles per gallon (on-road tailpipe) for cars and 38 miles per gallon (on-road tailpipe) by 2030. The Comprehensive scenario also models the

impact of federal policies aimed at removing inefficient vehicles from the road. Specifically, we assumed that for all existing passenger and freight vehicles, the average expected lifetime is reduced from 11-20 years (depending on the size classes) to 10 years, and that inefficient vehicles produced prior to 2010 are fully retired by 2030.

We further assume an accelerated transition toward electric vehicles in the Enhanced Non-Federal and comprehensive scenarios, using NREL's "Electrification Futures Study: Scenarios of Electric Technology Adoption and Power Consumption for the United States" as the basis for the potential for electrified light-, medium-, and heavy-duty vehicles and transit buses. For the Enhanced Non-Federal scenario, we assume that Tier 1 and 2 states pass policies and work with the private sector to accelerate electric vehicles such that EV sales rates of 66% for light-duty cars, 56% for light-duty trucks, 20% for medium-duty trucks, 60% for transit buses, and 15% for heavy-duty vehicles are achieved by 2030. For Tier 3 states, we assume sales in line with the NREL EFS medium scenario, including 63% of light-duty cars, 53% of light-duty trucks, 10% of medium-duty trucks, 3% of heavy-duty, and 20% of transit bus sales by 2030. Sales percentages were converted into passenger kilometers traveled or ton-kilometers using average annual vehicle miles traveled data from U.S. Federal Highway Administration (FHWA) Highway Statistics 2017[49] and ton-kilometer data from UC Davis.[50] For the Comprehensive scenario, we assume that sales rates in Tier 1 and 2 states are achieved nation-wide and that 100% of new bus sales nationwide are electric by 2030.

Hydrofluorocarbons

At the federal level, EPA passed rules in 2015 to phase out certain uses of HFCs through its Significant New Alternatives Policy (SNAP) program. However, the rules were vacated by the DC Court of Appeals in 2017[51] and so are not included in the Current Measures scenario. EPA also issued a rule in 2016 expanding refrigerant management practices to cover HFCs, which we assumed remains effective in the Current Measures scenario.[52]

At the state level, California began addressing refrigerant leaks in 2011 through its Refrigerant Management Program (RMP)[53] and adopted a regulation in 2018 to preserve the SNAP prohibitions within the state. To quantify the impact of this measure, we used California's estimates of the maximum impact of this regulation for years 2018 through 2030.[54] Washington and Vermont adopted similar policies in 2019. To estimate the impact of these actions, we assumed they achieve the California reduction levels, adjusted to their respective baselines.

For the Enhanced Non-Federal scenario, we assume that Tier 1 and Tier 2 states follow the lead of California, Washington, and Vermont by adopting SNAP programs that include aerosols as well as refrigerants. The projected impacts of these programs are derived from the above-mentioned analysis published by the California Air and Resources Board (CARB), with impacts adjusted to each state's baseline. We also assume that 50% of U.S. supermarkets achieve leakage reductions from refrigeration equipment in-line with average reduction levels currently achieved by EPA GreenChill partners. [55,56] These voluntary measures are conservatively assumed to overlap with other policies rather than being additional.

For the Comprehensive scenario, we assume that all states achieve a 40% reduction in HFC emissions by 2030 from 2013 levels, in-line with current best-in-class state proposals as well as the impact of federal ratification of the Kigali Amendment. Projected impacts of these policies were also derived from analysis conducted by CARB.[57]

Biofuels

For both the Enhanced Non-Federal and Comprehensive scenarios, we assume that the production and use of cellulosic biofuels and biodiesel continues to grow at historic rates of 30% and 10% annually, respectively. We also assume that the production and use of advanced biofuels such as sustainable aviation fuel (SAF) grows approximately 2% each year as new production facilities come online[58] and leading Tier 1 states and cities work with their large hub airports to encourage the blending of SAF with traditional jet fuel to achieve a 10% HEFA blend by 2030. Using data on jet

fuel consumption by state[59], and enplaned passengers per airport[60], we approximated jet fuel consumption per large hub airport to inform our advanced biofuels projections. We checked our biofuel production projections for consistency with the $60/ton base case of the 2016 Billion-Ton Report[61].

Industrial Emissions Reductions

We assume that in the Enhanced Non-Federal and Comprehensive scenarios, states and the federal government enact a suite of policies to reduce industrial GHG emissions. For the Enhanced Non-Federal scenario, Tier 1 states rapidly increase efficiency such that 75% of industrial businesses are required to adopt ISO 50001 energy efficiency protocols by 2030. Tier 2 states adopt this policy but only target half of industrial facilities, while Tier 3 states apply the standard to 10% of facilities. We assume that adoption of ISO 50001 results in a 5% efficiency gain in the first year of implementation and an additional 1% every year after that. This is on top of business-as-usual efficiency improvements of 1.2% percent per year. The Comprehensive scenario extends this policy to all 50 states such that, by 2030, 100% of industrial companies nationally have adopted ISO 50001.

In the Enhanced Non-Federal scenario, we apply the technology adoption assumptions from NREL's Electrification Futures "High Case" scenario to Tier 1 and 2 states and the "Medium Case" scenario for Tier 3 states. In the Comprehensive scenario, federal policies drive industrial electrification in all 50 states consistent with NREL's Electrification Futures "High Case" scenario, achieving a net decrease in energy consumption from industrial processes of 118 TBtu by 2030, while also increasing electricity share by 60 TBtu. We also model a policy representing the impact of state and federal policies on cement emissions. Using analyses from EFI,[62] McKinsey,[63] and IEA,[64] we assume in the Enhanced Non-Federal scenario that Tier 1 states reduce cement emissions by 22% from 2018 levels by 2030. For the Comprehensive scenario, we assume that a federal policy places additional requirements on Tier 2 and 3 states such that all states achieve the 20% reductions. We also assume that states promote industrial CCUS such that by 2030, 50 Mt CO_2e is sequestered in both the Enhanced Non-Federal and Comprehensive scenarios.

Land Use

EPA's estimate of carbon dioxide removals from the land sink has varied significantly over time, reflecting the high uncertainty in land sector estimates as well as continued methodological improvements. Projections are even more uncertain with EPA[65][66] and USDA models showing divergent estimates. Given this uncertainty, our Current Measures scenario assumes a constant sink through 2030 and estimate uncertainty about this sink using an adjusted range of high and low projections from the Biennial Report. [67] For the Enhanced Non-Federal scenario, we assume that states implement programs to promote conservation, restoration, and improved land management.[68] California, which has its own goals for natural and working lands, achieves -40 Mt CO_2e, and other Tier 1 States achieve 60% of additional mitigation potential available at $10/ton by 2030. Tier 2 states achieve 30% of potential. Combined, these actions increase the land sink by 79 Mt CO_2e in 2030. For the Comprehensive scenario, California achieves -40 Mt CO_2e and all other states achieve 60% of additional mitigation potential at $10/ton by 2030, increasing the land sink by 167 Mt CO_2e in 2030.

Other Methane and Nitrous Oxide

Methane from livestock, landfills, coal mining, and crops, and nitrous oxide from crops and nitric and adipic acid production also contribute to climate change. We used projected emissions for these gases from EPA in our Current Measures scenario.[69] In the Enhanced Non-Federal scenario, we assume that states regulate methane and nitrous oxide and achieve mitigation that is cost-effective but do not achieve any mitigation that has marginal costs, calculated using the EPA's non-CO_2 marginal abatement cost curves.[70] For the Comprehensive scenario, we assume that federal regulations are put in place such that all states achieve 100% of the potential for non-CO_2 mitigation accessible at $100/ton or less for livestock and $30/ton for landfill methane, coal mining, croplands, and nitric and adipic acid.

Supplementary References

[1] New Climate Institute, World Resources Institute, CDP, and The Climate Group. "Non-State and Subnational Action Guidance." Initiative for Climate Action Transparency, July 26, 2017. http://www.climateactiontransparency.org/wp-content/uploads/2017/07/ICAT-Non-State-and-Subnational-Action-Guidance-26-JUL-2017.pdf.
[2] Kovac, Alex, and Wee Kean Fong. "Compact of Mayors Emissions Scenario Model." Technical Note. World Resources Institute, December 2015. https://www.wri.org/sites/default/files/Compact_of_Mayors_Emissions_Scenario_Model.pdf.
[3] David Rich, Pankaj Bhatia, Jared Finnegan, Kelly Levin, Apurba Mitra. "Policy and Action Standard, an accounting and reporting standard for estimating the greenhouse gas effects of policies and actions" Greenhouse Gas Protocol. https://ghgprotocol.org/sites/default/files/standards/Policy%20and%20Action%20Standard.pdf
[4] Smart Electric Power Alliance (SEPA). Utility Carbon Reduction Tracker. https://sepapower.org/utility-carbon-reduction-tracker/
[5] U.S. Environmental Protection Agency, Natural Gas STAR Program. https://www.epa.gov/natural-gas-star-program/natural-gas-star-program
[6] Tom Land and U.S. Environmental Protection Agency (EPA). "GreenChill Annual Achievements." presented at the Energy & Store Development Conference, September 2017. https://www.epa.gov/sites/production/files/2017-09/documents/gc_recognition.presentation_2017_ceremony.pdf
[7] U.S. Energy Information Administration (EIA). Annual Energy Outlook 2019. https://www.eia.gov/outlooks/aeo/
[8] More detail on GCAM can be found at: http://jgcri.github.io/gcam-doc/toc.html. The version of GCAM-USA used in this model is based on GCAM X.X.
[9] Alvarez et al. 2018. "Assessment of methane emissions from the U.S. oil and gas supply chain." Science. July 13, 2018. https://science.sciencemag.org/ content/361/6398/186
[10] Bloomberg New Energy Finance, "New Energy Outlook 2018," https://bnef.turtl.co/story/neo2018.
[11] Nelson, W., and S. Liu. "Half of U.S. Coal Fleet on Shaky Economic Footing: Coal Plant Operating Margins Nationwide." Bloomberg New Energy Finance, 2018.
[12] U.S. Department of Energy. "Vogtle." https://www.energy.gov/lpo/vogtle
[13] Paul Picciano, Kevin Rennert, and Daniel Shawhan. "Projected Effects of the Clean Energy Standard Act of 2019." Resources for the Future." https://www.rff.org/publications/issue-briefs/projected-effects-clean-energy-standard-act-2019/
Full data from the modeling analysis was provided to America's Pledge offline.
[14] U.S. Energy Information Administration (EIA). Annual Electric Power Industry Report, Form EIA-861 detailed data files. https://www.eia.gov/ electricity/data/eia861/
[15] Charles Teplin, Mark Dyson, Alex Engel, and Grant Glazer. The Growing Market for Clean Energy Portfolios: Economic Opportunities for a Shift from New Gas-Fired Generation to Clean Energy Across the United States Electricity Industry. Rocky Mountain Institute, 2019, https://rmi.org/cep-reports
[16] Charles Teplin, Mark Dyson, Alex Engel, and Grant Glazer. "The Growing Market for Clean Energy Portfolios: Economic Opportunities for a Shift from New Gas-Fired Generation to Clean Energy Across the United States Electricity Industry". Rocky Mountain Institute, 2019, https://rmi.org/cep-reports.
[17] Lawrence Berkely Lab (LBL). Renewables Portfolio Standards Resources. https://emp.lbl.gov/projects/renewables-portfolio

---

[18] U.S. Energy Information Administration (EIA). Annual Electric Power Industry Report, Form EIA-861 detailed data files. https://www.eia.gov/electricity/data/eia861/

[19] State and Local Energy Data (SLED), August 2019. U.S. Department of Energy. https://www.eere.energy.gov/sled

[20] U.S. Energy Information Administration (EIA). Annual Electric Power Industry Report, Form EIA-861 detailed data files. https://www.eia.gov/electricity/data/eia861/

[21] Smart Electric Power Alliance (SEPA). Utility Carbon Reduction Tracker. https://sepapower.org/utility-carbon-reduction-tracker/

[22] Bloomberg New Energy Finance (BNEF). U.S. Plant Stack dataset.

[23] U.S. Environmental Protection Agency, "Natural Gas and Petroleum Systems in the GHG Inventory: Additional Information on the 1990-2016 GHG Inventory (published April 2018)," https://www.epa.gov/ghgemissions/natural-gas-and-petroleum-systems-ghg-inventory-additional-information-1990-2016-ghg.

[24] U.S. Energy Information Administration (EIA). Annual Electric Power Industry Report, Form EIA-861 detailed data files. https://www.eia.gov/electricity/data/eia861/

[25] U.S. Energy Information Administration (EIA). Natural Gas Consumption by End Use. https://www.eia.gov/dnav/ng/ng_cons_sum_a_EPG0_vgt_mmcf_m.htm

[26] ACEEE's State Energy Efficiency Scorecard ranks states on their efficiency policies and programs, assessing performance and also documenting best practices and recognizing leadership. Berg, Weston, Seth Nowak, Meegan Kelly, Shruti Vaidyanathan, Mary Shoemaker, Anna Chittum, Marianne DiMascio, and Heather DeLucia. "The 2017 State Energy Efficiency Scorecard." American Council for an Energy-Efficient Economy, September 2017. http://aceee.org/sites/default/files/publications/researchreports/u1710.pdf

[27] For more details, see: U.S. Environmental Protection Agency. 2014. "Background and Draft Methodology for Estimating Energy Impacts of EE/RE Policies." Accessible at: Synapse. 2012. "State Energy Efficiency in the AEO Electricity Forecasts." Accessible at https://www.synapse-energy.com/project/state-energy-efficiency-embedded-annual-energy-outlook-forecasts

This methodology is also consistent with the examples EPA provided to support Clean Power Plan compliance in its technical support document entitled "Incorporating RE and Demand-Side EE Impacts into State Plan Demonstrations." Accessible at: http://epa.gov/airquality/cpp/tsd-cppincorporating-re-ee.pdf

[28] American Council for an Energy-Efficient Economy (ACEEE). State and Local Policy Database. https://database.aceee.org/

[29] U.S. Department of Energy (DOE). State & Local Energy Data. https://www.eere.energy.gov/sled/#/

[30] Bruce Tonn, David Carroll, Erin Rose, Beth Hawkins, Scott Pigg, Daniel Bausch, Greg Dalhoff, Michael Blasnik, Joel Eisenberg, Claire Cowan, and Brian Conlon. "Weatherization Works II – Summary of Findings From the ARRA Period Evaluation of the U.S. Department of Energy's Weatherization Assistance Program." Oak Ridge National Laboratory, July 2015. https://weatherization.ornl.gov/wp-content/uploads/pdf/WAPRecoveryActEvalFinalReports/ORNL_TM-2015_139.pdf

[31] Michael Blasnik, Greg Dalhoff, David Carroll, Ferit Ucar, and Dan Bausch. "Evaluation of the Weatherization Assistance Program During Program Years 2009-2011 (American Recovery and Reinvestment Act Period): Energy Impacts for Single Family Homes." Oak Ridge National Laboratory, March 2015. https://weatherization.ornl.gov/wpcontent/uploads/pdf/WAPRecoveryActEvalFinalReports/ORNL_TM-2014_582.pdf

[32] Ibid.

---

[33] Ibid.
[34] RA Athalye, B Liu, D Sivaraman, R Bartlett, and DB Elliott. "Impacts of Model Building Energy Codes." Pacific Northwest National Laboratory, October 2016. https://www.energycodes.gov/sites/default/files/documents/Impacts_Of_Model_Energy_Codes.pdf
[35] Sierra Club. Ready for 100 Commitments. https://www.sierraclub.org/ready-for-100/commitments
[36] Climate Mayors. http://climatemayors.org/
[37] Chen, Yan; Liu, Bing; Zhang, Jian; Rosenberg, Michael; Edelson, Jim; Lyles, Mark. "Final Energy Savings Analysis of the Proposed NYStretch-Energy Code 2018." Pacific Northwest National Lab, December 31, 2018. DOI: 10.2172/1489815.
[38] National Renewable Energy Laboratory. Electrification Futures Study: Scenarios of Electric Technology Adoption and Power Consumption for the United States. https://www.nrel.gov/docs/fy18osti/71500.pdf
[39] American Council for an Energy-Efficient Economy (ACEE), https://database.aceee.org/.
[40] U.S. Department of Transportation Federal Highway Administration, "Highway Statistics Series Publications," https://www.fhwa.dot.gov/policyinformation/statistics.cfm.
[41] American Council for an Energy-Efficient Economy (ACEEE). https://database.aceee.org/.
[42] U. S. Department of Energy, Energy Efficiency & Renewable Energy, "State & Local Energy Data," https://apps1.eere.energy.gov/sled/#/.
[43] California Air Resources Board. 2019. Terms for Light-Duty Greenhouse Gas Emissions Standards. https://ww2.arb.ca.gov/sites/default/files/2019-07/Auto%20Terms%20Signed.pdf
[44] The California Low-Emission Vehicle Regulations. "§ 1962.2 Zero-Emission Vehicle Standards for 2018 and Subsequent Model Year Passenger Cars, Light-Duty Trucks, and Medium-Duty Vehicles." Accessed August 16, 2018. https://www.arb.ca.gov/msprog/zevprog/zevregs/1962.2_Clean.pdf.
[45] U.S. Energy Information Administration (EIA). Annual Energy Outlook 2019. https://www.eia.gov/outlooks/aeo/tables_ref.php
[46] U.S. Federal Highway Administration (FWHA). Highway Statistics 2017. https://www.fhwa.dot.gov/policyinformation/statistics/2017/mv1.cfm
[47] U.S. Energy Information Administration (EIA). Analysis of the Effect of Zero-Emission Vehicle Policies: State-Level Incentives and the California Zero-Emission Vehicle Regulations. https://www.eia.gov/analysis/studies/transportation/zeroemissions/pdf/zero_emissions.pdf
[48] American Council for an Energy-Efficient Economy (ACEEE). https://database.aceee.org/.
[49] U.S. Department of Transportation Federal Highway Administration, "Highway Statistics Series Publications". https://www.fhwa.dot.gov/policyinformation/statistics/2017/
[50] Mishra, Gouri Shankar, Page Kyle, Jacob Teter, Geoffrey M. Morrison, Sanling Kim, and Sonia Yeh." *Transportation module of global change assessment model (GCAM): model documentation*". No. UCD-ITS-RR-13-05. 2013.
[51] The Court of Appeals remanded Rule 20, stating that the EPA could not use the SNAP program to require businesses to replace HFCs with alternatives after already requiring them to replace ozone-depleting substances with HFCs. (*Mexichem Fluor v. EPA, https://www.cadc.uscourts.gov/internet/opinions.nsf/3EDC3D4817D618CF8525817600508EF4/$file/15-1328-1687707.pdf*)
[52] The EPA's Updated Refrigerant Management Requirements: What Supermarkets and Property and Facility Managers Need to Know.

---

https://www.epa.gov/sites/production/files/201609/documents/608_fact_sheet_supermarkets_property_managers_0.pdf
[53] Ibid.
[54] California Environmental Protection Agency, Air Resources Board. "Table B2. Reductions (MMT CO_2 E) each calendar year, shown by equipment production year for all emissions sectors covered by proposed regulation," Appendix B: Emission Estimates. https://www.arb.ca.gov/regact/2018/casnap/isorappb.pdf
[55] Tom Land and U.S. Environmental Protection Agency (EPA). "GreenChill Annual Achievements." presented at the Energy & Store Development Conference, September 2017. https://www.epa.gov/sites/production/files/2017-09/documents/gc_recognition.presentation_2017_ceremony.pdf.
[56] Ibid.
[57]Staff Report: Initial Statement of Reasons. State of California Air Resources Board. https://ww3.arb.ca.gov/regact/2018/casnap/isor.pdf
[58] "Alternative Aviation Fuels: Overview of Challenges, Opportunities, and Next Steps." U.S. Department of Energy. March 2017. https://www.energy.gov/sites/prod/files/2017/03/f34/alternative_aviation_fuels_report.pdf
[59] Energy Information Administration (EIA). 2018. "Jet fuel consumption, price, and expenditure estimates". 2018. https://www.eia.gov/state/seds/data.php?incfile=/state/seds/sep_fuel/html/fuel_jf.html
[60] Federal Aviation Administration. 2018. National Plan of Integrated Airport Systems (2019-2023). https://www.faa.gov/airports/planning_capacity/npias/reports/
[61] U.S. Department of Energy. 2016. 2016 Billion-Ton Report: Advancing Domestic Resources for a Thriving Bioeconomy, Volume 1: Economic Availability of Feedstocks. M. H. Langholtz, B. J. Stokes, and L. M. Eaton (Leads), ORNL/TM-2016/160. Oak Ridge National Laboratory, Oak Ridge, TN. 448p. doi: 10.2172/1271651. http://energy.gov/eere/bioenergy/2016-billion-ton-report
[62] Energy Futures Initiative. 2019. "Optionality, Flexibility and Innovation: Pathways for Deep Decarbonization in California". https://static1.squarespace.com/static/58ec123cb3db2bd94e057628/t/5ced6fc515fcc0b190b60cd2/1559064542876/EFI_CA_Decarbonization_Full.pdf
[63] McKinsey and Company. 2018. "Decarbonization of industrial sectors: the next frontier". https://www.mckinsey.com/~/media/mckinsey/business%20functions/sustainability/our%20insights/how%20industry%20can%20move%20toward%20a%20low%20carbon%20future/decarbonization-of-industrial-sectors-the-next-frontier.ashx
[64] International Energy Agency. 2018. "Technology Roadmap – Low-Carbon Transition in the Cement Industry". https://www.iea.org/newsroom/news/2018/april/cement-technology-roadmap-plots-path-to-cutting-co2-emissions-24-by-2050.html
[65] Tian, Xiaohui, Brent Sohngen, Justin Scott Baker, Sara Ohrel. 2018. "Will U.S. Forests Continue to Be a Carbon Sink". Land Economics: 94 (1). https://www.researchgate.net/publication/322669663_Will_US_Forests_Continue_to_Be_a_Carbon_Sink
[66] U.S. Department of Agriculture. 2016. USDA Integrated Projections for Agriculture and Forest Sector Land Use, Land-Use Change, and GHG Emissions and Removals: 2015-2060. https://www.usda.gov/oce/climate_change/mitigation_technologies/Projections2015documentation01192016.docx
[67] U.S. Department of State. 2016. Second Biennial Report of the United States of America. https://unfccc.int/files/national_reports/biennial_reports_and_iar/submitted_biennial_reports/application/pdf/2016_second_biennial_report_of_the_united_states_.pdf

---

[68] Nature 4 Climate. "US State Mapper." Accessed Sep 2019. https://nature4climate.org/u-s-carbon-mapper/
[69] EPA, "Global Non-CO2 Greenhouse Gas Emission Projections & Mitigation: 2015-2050." https://www.epa.gov/global-mitigation-non-co2-greenhouse-gases
[70] Ibid.
